# Metastable CdTe@HgTe Core@Shell Nanostructures Obtained by Partial Cation Exchange Evolve into Sintered CdTe Films Upon Annealing

**DOI:** 10.1021/acs.chemmater.9b05281

**Published:** 2020-03-12

**Authors:** Irene Rosina, Beatriz Martín-García, Davide Spirito, Zhiya Dang, Graziella Gariano, Sergio Marras, Mirko Prato, Roman Krahne, Luca De Trizio, Liberato Manna

**Affiliations:** † Nanochemistry Department, Istituto Italiano di Tecnologia (IIT), via Morego 30, 16163 Genova, Italy; ‡ Dipartimento di Chimica e Chimica Industriale, Università Degli Studi di Genova, Via Dodecaneso 31, 16146 Genova, Italy; § Graphene Labs, Istituto Italiano di Tecnologia (IIT), via Morego 30, 16163 Genova, Italy; ∥ Optoelectronics, Istituto Italiano di Tecnologia (IIT), via Morego 30, 16163 Genova, Italy; ⊥ Materials Characterization Facility, Istituto Italiano di Tecnologia (IIT), via Morego 30, 16163 Genova, Italy

## Abstract

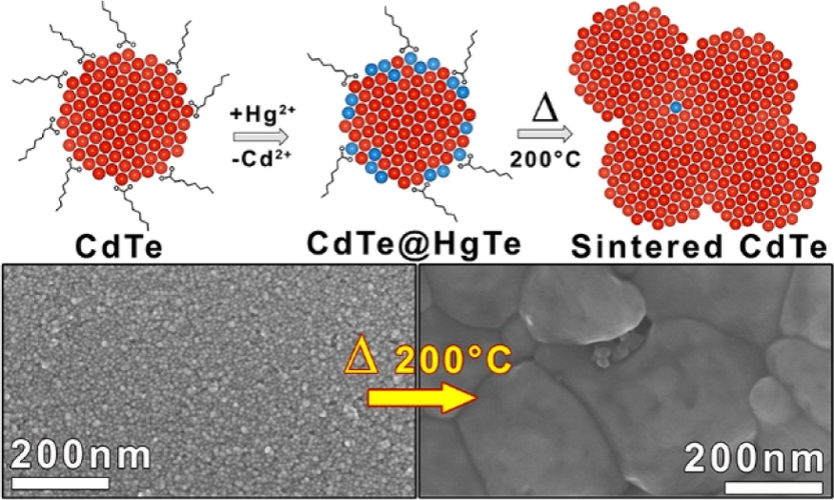

Partial Hg^2+^ → Cd^2+^ cation exchange (CE) reactions were exploited to transform colloidal CdTe nanocrystals (NCs, 4–6 nm in size) into CdTe@HgTe core@shell nanostructures. This was achieved by working under a slow CE rate, which limited the exchange to the surface of the CdTe NCs. In such nanostructures, when annealed at mild temperatures (as low as 200 °C), the HgTe shell sublimated or melted and the NCs sintered together, with the concomitant desorption of their surface ligands. At the end of this process, the annealed samples consisted of ligand-free CdTe sintered films containing an amount of Hg^2+^ that was much lower than that of the starting CdTe@HgTe NCs. For example, the CdTe@HgTe NCs that initially contained 10% of Hg^2+^, after being annealed at 200 °C were transformed to CdTe sintered films containing only traces of Hg^2+^ (less than 1%). This procedure was then used to fabricate a proof-of-concept CdTe-based photodetector exhibiting a photoresponse of up to 0.5 A/W and a detectivity of ca. 9 × 10^4^ Jones under blue light illumination. Our strategy suggests that CE protocols might be exploited to lower the overall costs of production of CdTe thin films employed in photovoltaic technology, which are currently fabricated at high temperatures (above 350 °C), using post-process ligand-stripping steps.

## Introduction

In the last few years, cation exchange (CE) reactions have been extensively studied as a versatile tool to transform colloidal nanocrystals (NCs) directly in solution or deposited as NC films.^1−8^ Through these reactions, either a fraction or all of the “host” metal cations of presynthesized NCs are replaced with new cations, while preserving both the NC size and shape and, in many cases, the crystal structure.^2^ Depending both on the miscibility of the reactant and of the product materials, and on the kinetics of the CE reaction, different types of nanostructures can be accessed: alloy NCs, doped systems, dimers (also termed as “Janus-like”), and core@shell (or core@graded-shell) heterostructures, the latter even with elaborate architectures (quantum wells, multiple-cores@shell, etc.).^2,9,10^ CE reactions, especially if performed at low temperatures, can even lead to metastable nanostructures (i.e., kinetically accessed).^2^ These structures, in turn, can be transformed to more stable ones if subjected to either e-beam irradiation or annealing.^2,10−16^ For example, different groups have demonstrated that core@shell structures prepared by partial CE reaction (i.e., Cu_2_Te@PbTe, Cu_2_Te@SnTe, PbSe@CdSe, etc.) transform, upon heating, to more thermodynamically stable configurations, such as the dimer structures in which the interface energy between the two materials is minimized.^10,14^

Among the different CE reactions involving metal chalcogenide NCs, the CdTe → HgTe transformation is of particular interest as it leads to NCs having a bandgap ranging from 1.6 eV (bulk CdTe) to ∼0 eV (bulk HgTe), and is, thus, of relevance for IR sensing, IR detection/imaging, and for photovoltaics.^17−21^ CdTe and HgTe are completely miscible materials as both have a zinc blende crystal structure, with lattice parameters being 6.48 and 6.46 Å, respectively, hence with a lattice mismatch of only 0.3%. Alloy Cd_1–*x*_Hg_*x*_Te NCs, prepared from CdTe NCs by partial exchange of Cd^2+^ with Hg^2+^ ions, have a photoluminescence (PL) emission that can be tuned from ∼500 to ∼1100 nm.^18−20,22−26^ Such alloy NCs form when the partial CE reaction is performed under thermodynamic control, that is, by assuring a fast CE rate or a long reaction time.^18,22^ This can be achieved by employing highly reactive Hg precursors,^23^ alkylamines (which preferentially bind Cd^2+^ cations and favor their extraction from the NCs)^19,23,27^ and by working at high temperatures (i.e., 150 °C).^27^ On the other hand, at a low rate of partial CE, the product consists of CdTe@HgTe core@thin-shell NCs. Experimentally, this has been realized by working at room temperature (RT), using polar solvents and slow reacting Hg precursors [i.e., Hg(ClO_4_)_2_ or HgCl_2_], and by avoiding ligands that favor the extraction of Cd^2+^ ions.^20,23,26,28,29^ The formation of such heterostructures was explained by considering that the CdTe → HgTe exchange occurs through a fast surface exchange, followed by a much slower interdiffusion of the Hg^2+^ ions into the core of the NCs.^20,22,24,26,28^ This phenomenon is particularly evident when executing the reaction on CdTe nanoplatelets, in which the exchange with Hg^2+^ ions was found to be self-limited to the first surface monolayer: this led to the formation of CdTe@HgTe core@shell architectures when working with nanoplatelets that were more than three monolayers thick.^29−31^

**Scheme 1:**
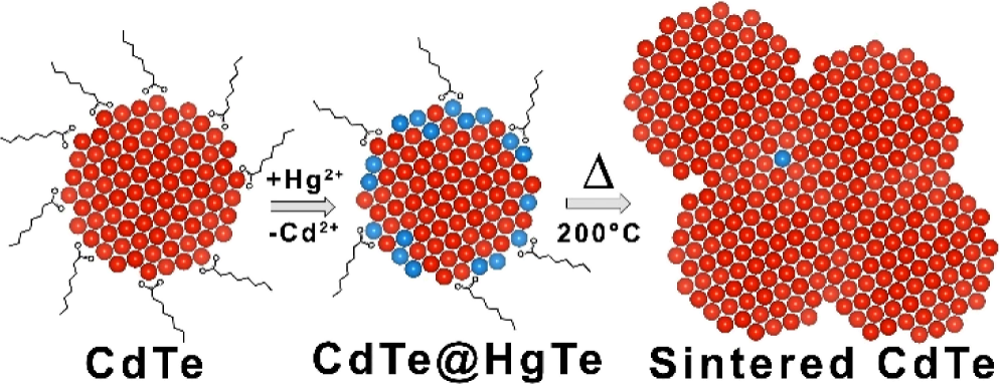
CE Reaction between CdTe NCs and Hg^2+^ Cations, Yielding CdTe@HgTe NCs The latter evolve into sintered CdTe films upon mild annealing at 200 °C.

Although strategies to prepare such CdTe@HgTe NCs and their related optical properties have been investigated in detail,^23^ not much is known about their thermal stability. To delve into this topic, in this work, we have focused our attention on the products of the partial CE between CdTe NCs (4–6 nm in size) and Hg^2+^ cations performed at a slow rate and their structural and morphological evolution under annealing. A short summary of the experiments is described here. Three samples containing 10, 30, or 40% of Hg (atomic percentage with respect to Cd) were prepared by adding sub-stoichiometric amounts of a slowly reacting Hg^2+^ precursor (i.e., HgCl_2_)^29^ dissolved in methanol to a dispersion of CdTe NCs in toluene at RT. The exchange under such conditions occurred preferentially at the surface of the CdTe NCs, yielding CdTe@HgTe core@shell NCs. Such nanostructures were found to be thermally unstable, as they underwent the following transformations when annealed at mild temperatures (as low as 200 °C): the HgTe external layer sublimated (or melted) and the residual cores sintered together, with the concomitant removal of the native surface ligands (i.e., the ligands used for their synthesis and bound to their surface, see Scheme 1). The final products of the annealing process consisted of ligand-free CdTe sintered films containing only a minor residual amount of Hg^2+^ if compared to that of the starting CdTe@HgTe NCs. Noticeably, the CdTe@HgTe samples that initially contained 10% of Hg^2+^ were transformed into sintered CdTe NCs containing only traces of Hg^2+^ (less than 1%).

CdTe is a material of great relevance in thin-film photovoltaics technology,^32,33^ and strategies are being developed in many groups to lower the costs of film fabrication, for example by solution approaches based on colloidal NC inks. However, to produce bulk-like films from such inks, the deposited NC films need to be annealed at temperatures above 350 °C in order to sinter the NCs together and to remove the ligands.^34−39^ By leveraging on our Cd^2+^ → Hg^2+^ exchange protocol, we could prepare CdTe sintered films using much milder annealing conditions (200 °C). These films exhibited a photoresponse up to 0.5 A/W and a detectivity of ca. 9 × 10^4^ Jones, in line with the CdTe NC film systems reported in the literature,^40−43^ highlighting the potential use of our strategy in the fabrication of future CdTe-based optoelectronic devices.

## Experimental Section

### Materials

Cadmium oxide (CdO) (99.999%) and Te powder (99.999%) were purchased from Strem Chemicals. Mercury(II) chloride (HgCl_2,_ 99.99%), oleic acid (OA, 90%), trioctylphosphine (TOP, 90%), tributylphosphine (97%), triethylphosphine (1.0 M in tetrahydrofuran) solution, octadecene (ODE, 90%), isopropanol (IPA, 99.8%), toluene (>99.5%, anhydrous), toluene (>99.5%), acetone (>99.5%), ethanol (≥99.8%, anhydrous), and Alconox detergent were purchased from Sigma-Aldrich. All chemicals were used without any further purification.

### Preparation of the Te Stock Solution

A Te precursor stock solution (10% Te by weight, 0.7 M) was prepared in a three-neck flask by dissolving 1.1 g of Te powder (8.6 mmol) in 12 mL (26.9 mmol) of TOP under inert atmosphere. The solution was heated up to 250 °C until the Te powder was fully dissolved. After cooling it down to RT, its color turned to straw-yellow. The solution was further degassed under vacuum for 30 min.

### Synthesis of CdTe NCs

The synthesis was adapted from the work of Zhang et al.^34^ In brief, 7.5 mmol CdO (960 mg), 30 mmol OA (9.5 mL), and ODE (10 mL) were loaded in a 50 mL flask and degassed at 110 °C for 1 h. Subsequently, the mixture was heated up to 300 °C under Ar in order to get an optically clear solution (indicating the formation of cadmium oleate complexes). This was followed by a further degassing step under vacuum at 110 °C to remove the water generated by the reaction. At this point, the flask was heated up to 280 °C, followed by the quick injection of TOP-Te (2.86 mL) diluted with 1 mL of ODE. After 10 s from the injection, the heating mantle was removed and the flask was quickly cooled to RT. The resulting NCs were collected by twofold precipitation with anhydrous ethanol from their colloidal solutions in anhydrous toluene followed by dissolution in toluene. The final purified NCs were redispersed in toluene at a concentration of 0.2 M in Cd.

### Partial CdTe → HgTe CE Reaction

The CE reactions were performed by following the procedure reported by Miszta et al.^44^ In detail, a dispersion of CdTe NCs in toluene ([Cd] = 5 mM) was mixed with a solution of HgCl_2_ in methanol (0.1 M) at RT under nitrogen and stirred for 1 min. The reaction was quenched by adding 10 mL of toluene. The NC product was cleaned twice by dispersion in toluene and precipitation by the addition of ethanol. If necessary, in the first cleaning step, OA (50 μL) was added to increase the colloidal stability of the resulting NCs. The precipitation was achieved by mild centrifugation (3000 rpm, 5 min). Different Hg/Cd feeding molar ratios were tested, ranging from 0.1 to 0.4.

### UV–Vis Absorption and PL Measurements

The UV–visible absorption spectra of both NCs in solution and NC films were recorded using a Varian Cary 5000 UV–vis–near-infrared (NIR) spectrophotometer (Agilent Technologies). The steady-state PL spectra of NCs in solution were collected on an Edinburgh Instruments FLS920 spectrofluorometer by exciting the samples with a xenon lamp at 450 nm. NC samples were dispersed in toluene and measured in quartz cuvettes with a path length of 1 cm.

### Raman Spectroscopy

The Raman spectra were acquired with a Renishaw inVia instrument, equipped with a 50× (0.75 N.A.) objective with an excitation wavelength of 532 nm with power <0.1 mW to avoid damaging the samples. For these measurements, the NC films were deposited onto SiO_2_/Si substrates.

### Inductively Coupled Plasma Optical Emission Spectroscopy Analysis

The elemental analysis was carried out *via* inductively coupled plasma optical emission spectroscopy (ICP–OES) on an iCAP 6000 Series ICP–OES spectrometer (Thermo Scientific). In a volumetric flask, each sample was dissolved in aqua regia [HCl/HNO_3_ 3:1 (v/v)] and left overnight at RT to completely digest the NCs. Afterward, Milli-Q grade water (18.3 MΩ cm) was added to the sample. The solution was then filtered using a polytetrafluorethylene membrane filter with 0.45 μm pore size. All chemical analyses performed by ICP–OES were affected by a systematic error of about 5%.

### Transmission Electron Microscopy Characterization

The samples were prepared by dropping dilute solutions of CdTe or CdTe@HgTe NCs onto carbon film-coated 400-mesh copper grids. For the transmission electron microscopy (TEM) analysis of annealed NCs, the grids onto which CdTe@HgTe NCs were deposited were annealed at 200 °C for 40 s under N_2_. Low-resolution TEM measurements were performed on a JEOL-1100 transmission electron microscope operating at an acceleration voltage of 100 kV. High-resolution TEM (HR-TEM) and high angular annular dark field-scanning TEM (STEM)–energy dispersive X-ray spectroscopy (EDS) analyses were performed on a JEOL JEM-2200FS microscope equipped with a Schottky emitter operated at 200 kV, a CEOS spherical aberration corrector of the objective lens, and an in-column energy filter (Omega-type), as well as Bruker QUANTAX 400 EDS system.

### Scanning Electron Microscopy and EDS Characterization

Scanning electron microscopy (SEM) images were acquired with a JEOL JSM-7500LA microscope equipped with a cold field-emission gun, operating at 15 kV acceleration voltage. EDS (Oxford Instrument, X-Max, 80 mm^2^) was used to evaluate the elemental ratios. All experiments were done at 8 mm working distance, 15 kV acceleration voltage, and 15 sweep count for each sample.

### X-ray Diffraction Characterization

The X-ray diffraction (XRD) analysis was performed on a PANalytical Empyrean X-ray diffractometer, equipped with a 1.8 kW Cu Kα ceramic X-ray tube and a PIXcel^3D^ 2 × 2 area detector, operating at 45 kV and 40 mA. Specimens for the XRD measurements were prepared by drop-casting a concentrated NC solution onto a quartz zero-diffraction single-crystal substrate. The diffraction patterns were collected under ambient conditions using parallel beam geometry and the symmetric reflection mode. XRD data analysis was carried out using the HighScore 4.1 software from PANalytical. High-temperature XRD analysis from RT to 350 °C, under inert atmosphere (N_2_), was performed using a Rigaku Smartlab system equipped with a 9 kW Cu Kα rotating anode (operating at 40 kV and 150 mA) and an Anton Paar DHS 900 domed hot stage. Samples for the measurements were prepared by dropcasting a concentrated NC solution onto a silicon wafer in a N_2_ filled glovebox.

### Fourier Transform Infrared Spectroscopy

Samples were weighted and mixed with KBr powder in a proportion of NCs/KBr = 0.5 mg:50 mg (1% w/w) and ground with a pestle in an agate mortar. The mixture was put in a die and pressed for 3 min with 3 tons producing 12 mm diameter pellets, which were analyzed by an Equinox 70 FT-IR, Bruker VERTEX Fourier transform infrared (FTIR) spectrometer. All spectra were recorded in the range from 3800 to 600 cm^–1^ with a resolution of 4 cm^–1^, accumulating 128 scans. A KBr pellet was used as blank.

### X-ray Photoelectron Spectroscopy Characterization

Measurements were performed on a Kratos Axis Ultra DLD spectrometer, using a monochromatic Al Kα source (15 kV, 20 mA). The photoelectrons were detected at a take-off angle of ϕ = 0° with respect to the surface normal. The pressure in the analysis chamber was maintained below 7 × 10^–9^ Torr for data acquisition. The data was converted to the VAMAS format and processed using the CasaXPS software, version 2.3.17. The binding energy (BE) scale was internally referenced to C 1s peak (BE for C–C = 284.8 eV).

### Device Fabrication and Photoconductivity Tests

The glass substrates were cleaned in an ultrasonic bath, first with acetone, then with IPA (8 min each step), and finally dried with N_2_ flow. A subsequent N_2_ plasma treatment was carried out. Homogeneous NC films were prepared by spin coating either (i) CdTe@HgTe or (ii) CdTe NC solutions ([Cd] = 0.2 mmol/mL) in toluene, in both cases *via* the layer-by layer deposition route. For the CdTe@HgTe NC case, the solution was deposited onto the substrate already mounted in the spin coater, and after 30 s the sample was spun at 800 rpm for 10 s and 3200 rpm for 40 s. For the CdTe NC case, the NC films were first deposited by spin coating (as described above), and subsequently, they were dipped for 2 s in a methanolic solution of HgCl_2_ (0.001 M). The resulting exchanged films were rinsed with IPA and then put on a hot plate at 100 °C for 2 min. In both cases, the following step consisted of annealing the films under inert N_2_ for 40 s at 200 °C on a hotplate to induce the NC sintering. This process was repeated three times to produce CdTe films of a desired thickness (ca. 400 nm), which was determined by a profilometer (Veeco Dektak 150). Metal deposition (Au, 80 nm) was carried out by e-beam evaporation (Kenosistec e-beam evaporator) with a shadow mask to define 1 mm × 1 mm pads separated by ca. 100 μm. The sample was kept at 20 °C during the metal deposition to avoid any additional annealing effects.

The electrical characterization of the films was performed at RT under air in a probe station from Janis Research in a two-probe configuration. The measurements were controlled by a PC using LabView. Illumination was provided through an optical window using fiber-coupled laser diodes (Thorlabs M455L4-royal blue at 455 nm, M505L4-cyan at 530 nm, and M625L4-red at 625 nm) and focused by external lenses on the sample to a spot size of approximately 1 mm. The spot position was controlled by translational stages. A mechanical shutter was used to block the light passing through the window for dark measurements. Before measuring the photoresponse, current–voltage (*I*–*V*) characterization in the dark was performed. The photoresponsivity was assessed for photoexcitation intensities ranging from 3 to 2000 mW cm^–2^ for continuous and modulated illumination with a Keithley 2600 SMU.

## Results and Discussion

**Figure 1:**
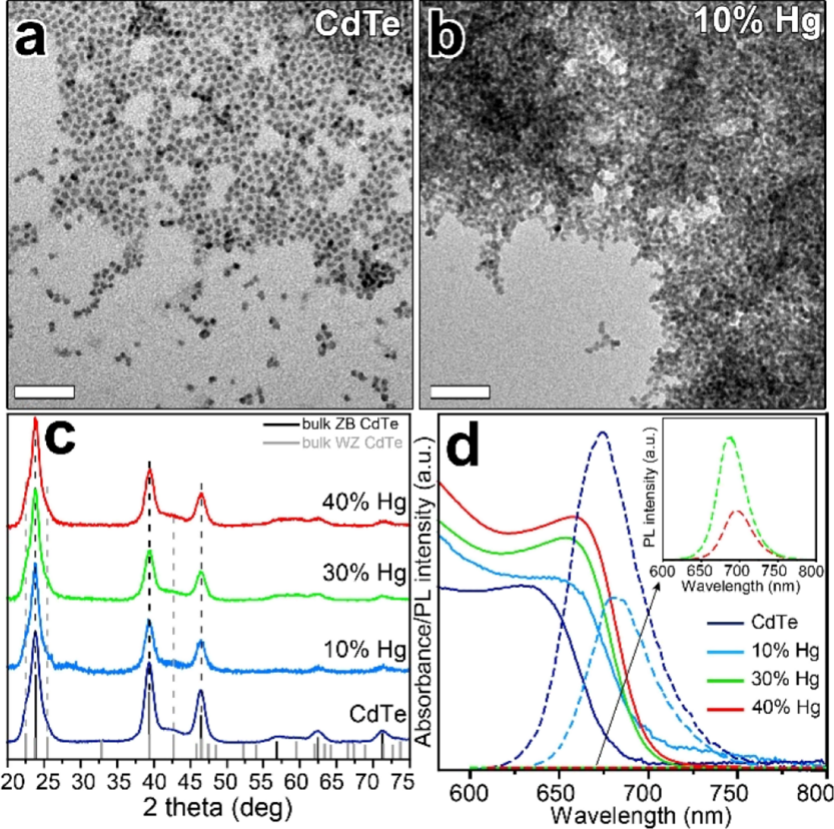
TEM images of (a) CdTe and (b) CdTe–Hg 10% NCs. (c) XRD patterns of CdTe and CdTe–Hg samples together with the bulk reflections of WZ (ICSD numbers 620518) and ZB (ICSD number 43712) CdTe structures. (d) Absorption and PL spectra of CdTe NCs before and after partial CE with Hg (λ_ex_ = 450 nm). The inset shows a magnification of the PL spectra of CdTe–Hg 30 and 40% samples.

Spherical CdTe NCs, with a mean diameter of 4.5 ± 1 nm, were synthesized following the procedure reported by Zhang et al.^32^ (see Figure 1a). The elemental analysis, conducted *via* ICP-OES, revealed that the composition of the NCs was nearly stoichiometric (i.e., CdTe), and the XRD characterization indicated the presence of a mixture of both CdTe wurtzite (WZ) and zinc blende (ZB) structures (ICSD numbers 620518 and 43712, respectively) (Figure 1c, blue pattern and Table 1). These NCs were used in partial CE reactions with Hg^2+^ cations in order to study the corresponding products and their evolution under annealing. The CdTe NCs were exposed to different Hg/Cd feed molar ratios, ranging from 0.1 (10%) to 0.4 (40%) employing a slowly reacting Hg precursor (i.e., HgCl_2_)^29^ dissolved in methanol, with the aim to lower the CE reaction rate and to limit the exchange to the surface of the NCs. For the same reason, and to avoid any possible reduction of Hg^2+^ to metallic Hg,^24,45^ we did not use any extra ligands, such as alkylamines, which are commonly utilized for this specific type of CE.^19,29−31^ Our CE reaction was, thus, driven only by the higher solubility of CdTe with respect to that of HgTe in polar solvents [*K*_SP_(HgTe) < *K*_SP_(CdTe)].^2,18,20,26,28^

**Table 1:** Composition and Size of Starting CdTe NCs and CdTe–Hg NCs before and after the Annealing Treatment at 200 °C

	pre-annealing		post-annealing	
Hg/Cd feed ratio (%)	stoichiometry^a^	size (nm)^b^	stoichiometry^a^	size (nm)^b^
	CdTe	4.2	CdTe	5.6
10	Cd_0.9_Hg_0.1_Te	6.2	Cd_0.9_Hg_0.01_Te	19.6
30	Cd_0.7_Hg_0.3_Te	5.8	Cd_0.9_Hg_0.1_Te	15.5
40	Cd_0.6_Hg_0.35_Te	6.2	Cd_0.9_Hg_0.3_Te	15.2

a Measured via ICP–OES.

b Calculated via the Scherrer formula.

The products of these partial CE reactions, which will be named CdTe–Hg 10, 30, and 40%, were NCs retaining the size and shape of the parental CdTe NCs, as revealed by TEM (Figures 1b and S1 of the Supporting Information). The ICP elemental analysis indicated that the extent of the Cd-for-Hg substitution was essentially in line with the Hg/Cd feed molar ratios employed (Table 1 and Figure S2). The X-ray photoelectron spectroscopy (XPS) measurements further confirmed the results of the ICP elemental analysis, additionally evidencing that Hg was present only in the +2 oxidation state (Hg 4f_7/2_ peak position at 100.6 eV), thus excluding the possible presence of metallic Hg (Figure S3). According to XRD analyses, the CE reaction did not induce any phase transition in the NCs because the same ratio of ZB to WZ phases as in the initial CdTe NCs was found in the products (see Figure 1c).

The starting CdTe NCs were characterized by an excitonic peak at 631 nm and a PL peak at 674 nm with a full width at half maximum of 40 nm (Figure 1d). The absorption and PL emission of the CdTe–Hg NCs systematically red-shifted with the increase of Hg in the NCs, while retaining the same linewidth (Figure 1d). Such a small red shift in both PL and absorbance indicated that partial CE did not lead to the formation of alloy NCs (i.e., Cd_1–*x*_Hg_*x*_Te), whose PL emission would have been expected at much longer wavelengths (i.e., 720 nm for Cd_0.9_Hg_0.1_Te and 820 nm for Cd_0.7_Hg_0.3_Te NCs of 4 nm).^19,23^ Instead, the products of the reaction were CdTe@HgTe core@shell NCs, similar to what was shown by Smith et al.^22^ Also, the more HgCl_2_ was added to the reaction (hence, more Hg was incorporated in the NCs), the more the PL intensity of the samples dropped. Compared to the starting CdTe NCs, the PL intensity of the CdTe–Hg 10% NC sample was reduced by ∼50%. In the CdTe–Hg 30 and 40% samples, the PL was almost quenched (Figure 1d), with no appearance of any extra NIR PL peak (up 1600 nm). This observation is consistent with the hypothesis of a shell growth (over that of an alloy formation); as revealed by Smith et al., the growth of a HgTe shell over a CdTe core leads to the spatial separation of photogenerated carriers and to a lowering of the oscillator strength of the 1st exciton peak.^22^ Therefore, a thicker HgTe shell would result in more efficient quenching of the PL from the CdTe core.

In order to estimate the thickness of the HgTe shell, which, given the small size of the NCs under analysis, could not be visualized by HRTEM imaging (Figures S6 and S7), we built a crystal model and we calculated that the expected Hg/Cd ratio to form a single HgTe monolayer on top of a 4.5 nm CdTe NC would be 0.42 (Figure S4). Our calculations, thus, indicated that the extent of the HgTe shell in our samples is below one monolayer, explaining, thus, the absence of HgTe XRD peaks even in the CdTe–Hg 40% sample.

We studied the stability of these NCs by drop-casting them onto a Si substrate and by exposing the resulting film to thermal treatment (under N_2_) from RT to 200 °C while monitoring their structural and compositional evolution. The XRD patterns acquired after the thermal treatment evidenced that all CdTe–Hg samples underwent sintering upon heating and that the ZB was preferentially stabilized over the WZ phase (Figure S5). In details, the size of the crystallites of the CdTe–Hg samples, calculated by employing the Scherrer formula, increased from 4–6 to 15–20 nm (Table 1 and Figure S5). In addition, the corresponding ICP elemental analyses revealed that such sintering was accompanied by a loss of Hg (see Table 1). In this regard, among the three CdTe–Hg samples, the CdTe–Hg 10% exhibited the largest extent of sintering (from 6.2 to 19.6 nm) and the lowest Hg content at the end of the process (less than 1%, see Table 1).

**Figure 2:**
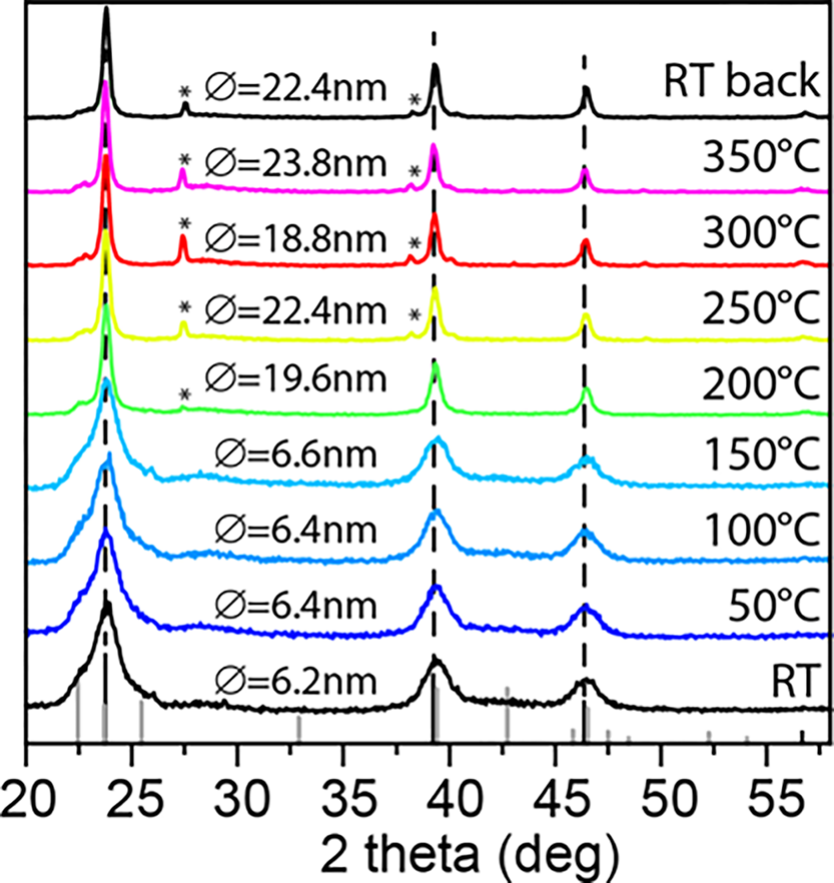
XRD patterns of CdTe–Hg 10% NCs subjected to thermal treatment under N_2_ from RT to 350 °C and then back to RT (RT-back). The bulk reflections of WZ and ZB CdTe structures are reported by means of gray and black bars, respectively. The metallic Te peaks are marked with asterisks. The diameter of the crystallites reported above each curve was calculated with the Scherrer formula.

Motivated by these findings, we investigated more closely the effects of thermal treatment on the CdTe–Hg 10% NCs by extending the heating range from RT to 350 °C (see Figure 2). The XRD patterns, acquired during such experiments, highlighted that most of the sintering occurred already at 200 °C, with not much additional evolution when going from 200 to 350 °C because in that temperature range, the mean grain size increased only from 19.6 to 22.4 nm (Figure 2). It was additionally observed that, in the 250–350 °C range, the sintering process was accompanied by the formation of metallic Te (Figure 2). At the same time, the ICP analysis revealed that the amount of Hg decreased from 1% at 200 °C to below the detection limit at 350 °C. Overall, these results were quite remarkable, considering that to sinter a film of colloidal CdTe NCs, one would typically require annealing temperatures above 350 °C.^32−39,46−48^ Indeed, as a control, we observed that a non-exchanged CdTe NC sample annealed at 200 °C did not undergo any appreciable sintering (Table 1 and Figure S5).

**Figure 3:**
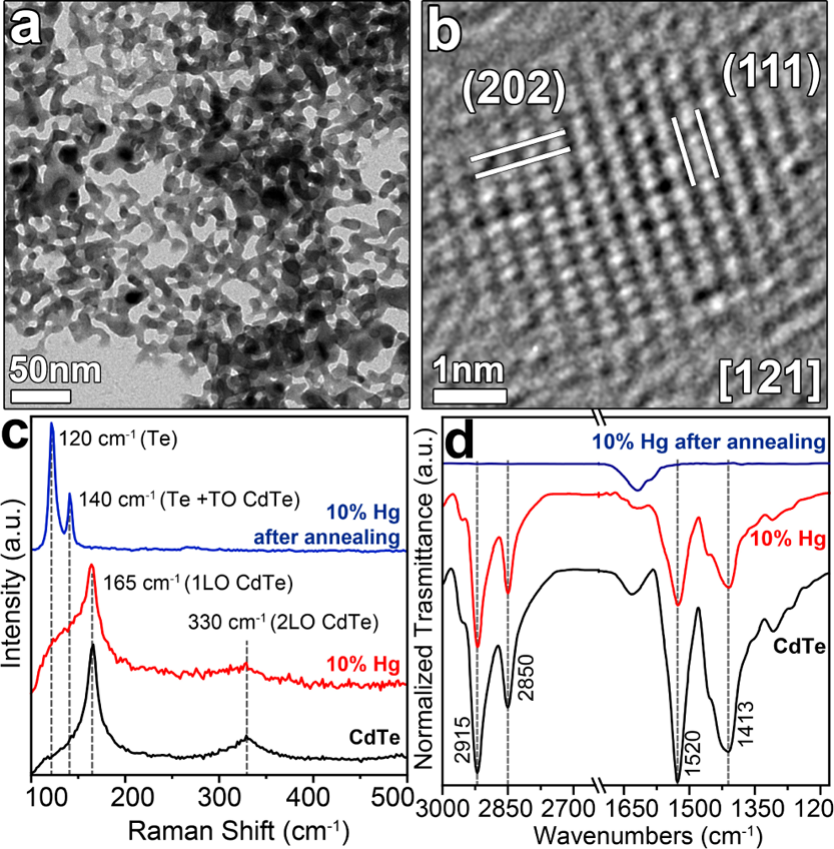
(a) TEM and (b) HRTEM images of CdTe–Hg10% NCs after annealing at 200 °C for 40 s. (b) Lattice fringes of the NCs could be indexed with the ZB CdTe structure. (c) Raman and (d) FTIR spectra of CdTe NCs (black curves), and CdTe–Hg 10% NCs before (red curves) and after (blue curves) annealing. In the Raman spectra, the modes corresponding to CdTe and metallic Te are pointed out to indicate the material evolution after the CE and subsequent sintering at 200 °C. In the FTIR spectra, the bands related to the carboxylic group (COO^–^) and the CH_2_ stretching of OA are indicated. These bands disappeared upon annealing (200 °C), indicating the absence of ligands at the end of the thermal treatment.

In order to better understand the thermal evolution of our CdTe–Hg 10% NCs, we carried out additional characterizations via HRTEM, STEM–EDS, and XPS analyses. The starting CdTe–Hg 10% NCs were characterized by a mixture of WZ and ZB structures (see Figure S6a) and a Hg content of 10% (see Figure S8), confirming our XRD and elemental analyses. The same NCs were annealed at 200 °C under N_2_ directly onto the carbon support of the TEM grid and were then re-examined under TEM (see the details in the Experimental Section); the resulting NCs had fused together, forming a continuous network (Figures 3a, S6b,c and S7). Such network was composed of NCs exhibiting fringes compatible with the CdTe ZB structure, in agreement with the XRD analyses, with no presence of Hg (i.e., the amount of Hg was below the detection limit of our EDS setup, see Figures 3b, S6, S7, and S9). The XPS analysis of the annealed CdTe–Hg 10% sample indicated the presence of Te, Cd, and Hg in −2, +2, and +2 oxidation states, respectively, with the amount of Hg being around 1% (Figure S3).

The Raman spectrum of the starting CdTe NCs deposited on silicon substrates (see the Experimental Section for further details) was characterized by bands at 166 and 329 cm^–1^ which correspond to the longitudinal optical (LO) and 2LO CdTe modes (Figure 3c).^23,49^ After the exchange with Hg^2+^ cations, a new broad band at lower wavenumbers to the LO-CdTe phonon modes (i.e., about at 138 cm^–1^) appeared and was assigned to the HgTe modes.^23,50^ After annealing at 200 °C, the modes at 166 and 329 cm^–1^ disappeared and two peaks emerged at 120 and 141 cm^–1^, which were attributed to the formation of metallic Te and the TO-CdTe phonon modes, in agreement with what emerged from the XRD analysis (see the Supporting Information for reference Raman spectra of Te, Figures S10 and 2).^51,52^

To analyze the effect of thermal treatment on the organic ligands that coated the surface of the NCs, we performed FTIR analyses on both the unexchanged OA-capped CdTe NCs and the CdTe–Hg 10% NCs before and after the annealing at 200 °C. Both samples were characterized by FTIR peaks at ∼1520 and 1413 cm^–1^, which could be ascribed to the asymmetric and the symmetric stretching modes of the COO^–^ group, and 2915 and 2850 cm^–1^ corresponding to the CH_2_ stretching of the OA. All these bands disappeared in the final annealed sample, indicating the absence of ligands in this sample (see Figure 3d).^34^ The broad band peak at 1600 cm^–1^, characterizing all samples, was due to the residual water inside the KBr matrix employed in FTIR analyses (Figure S11).

According to the experiments, the following picture can be drawn: upon heating at 200 °C or above, the starting CdTe–Hg NCs, instead of simply evolving into alloy Cd_1–*x*_Hg_*x*_Te NCs, underwent a substantial loss of Hg and Te [most likely by sublimation or melting of the HgTe superficial domain(s)]^21^ with the concomitant loss of surfactants and sintering of the NCs. In detail, when the amount of Hg inside the CdTe–Hg NCs was sufficiently low (i.e.,10%) and the annealing temperature was as low as 200 °C, the final sintered film had essentially a CdTe composition, or, in other words, nearly all initial Hg was expelled. On the other hand, when the amount of Hg inside the starting CdTe–Hg NCs was higher (above 30%), the film after annealing had a Cd_1–*x*_Hg_*x*_Te composition. In this case, not all HgTe had been able to sublimate or melt and part of the Hg had diffused into the film forming, most likely, an alloy. In all cases, part of the extracted Te recrystallized in the form of metallic Te. The sublimation or melting of the HgTe shell could be explained by considering that: (i) HgTe NCs of 9 nm size have been observed to undergo sintering at temperatures as low as 100 °C (the sintering was particularly pronounced at 150 °C), highlighting the softness of the HgTe lattice;^21^ (ii) in our systems, the expected thickness of HgTe domains should be below one monolayer, thus having a drastic melting-point depression.^53^

**Figure 4:**
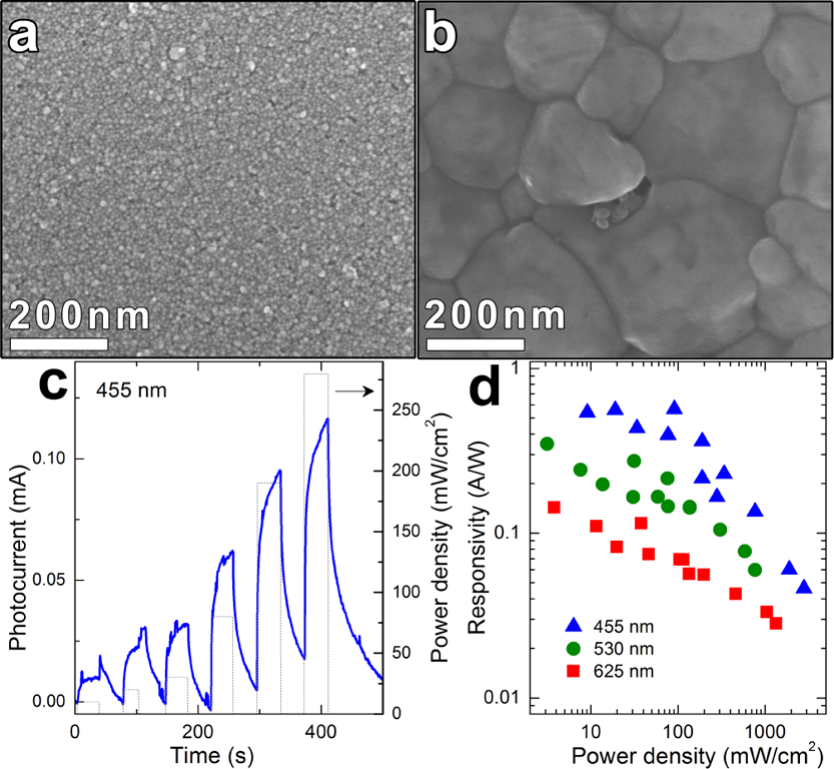
SEM images of a CdTe layer film before (a) and after sintering (b). (c) Time trace of the photocurrent (dark current at *t* = 0 s, left *y*-axis) while the light was switched on and off, at RT in air under 455 nm excitation at different power density (right *y*-axis). (d) Responsivity as a function of power density at different photoexcitation wavelengths: 455 nm (blue), 530 nm (green), and 625 nm (red). The on/off signal was allowed to stabilize for 20 s for this evaluation.

**Scheme 2:**
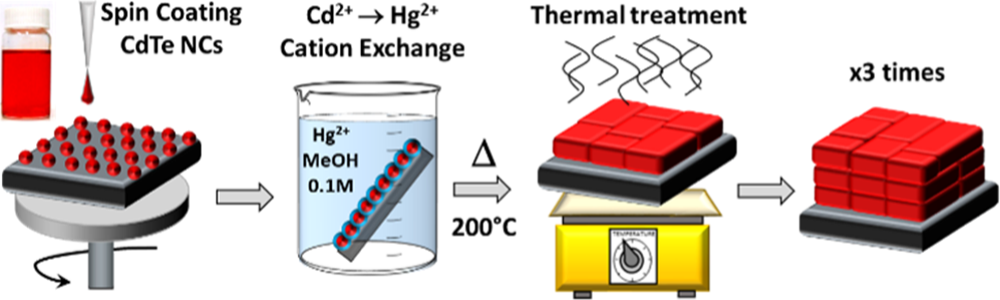
Sintered CdTe Thin Film Preparation Starting from CdTe NCs

Prompted by these results, we decided to exploit our strategy to fabricate and test a proof-of-concept CdTe thin-film-based photodetector device (see the Experimental Section for details on device fabrication). Initially, we employed an ink of CdTe–Hg 10% NCs; however, with this material, we did not obtain homogeneous CdTe films (see Figure S12a,b). We attribute this to the fact that, after the CE, the resulting CdTe–Hg NCs lost colloidal stability, forming NC dispersions that were cloudy (due to the presence of aggregates). Indeed, the responsivity (*R*) of the device prepared with the sintered CdTe–Hg 10% NCs reached only ca. 7 × 10^–4^ A/W under white light (see Figure S12c). In order to improve the quality of the films, we also devised a different strategy, consisting of first depositing CdTe NCs to form homogeneous films, followed by CE directly on the films. In detail, we prepared 3-layer sintered films by first spin coating the CdTe NCs, and then by dipping the resulting film into a solution of Hg^2+^ cations in methanol (achieving the desired extent of the Cd → Hg CE, i.e., 10% of Hg) and, eventually, by annealing the exchanged film at 200 °C for 40 s (Figure S13). This process was repeated three times (see Scheme 2). The morphology and composition of each CdTe layer and of the final layer stack was characterized by SEM analysis, which confirmed our previous results: upon annealing at 200 °C, the NCs film, initially composed of 4.2 nm NCs (Figure 4a), underwent sintering with the formation of large grains, in this case, having a mean size of hundreds of nm (Figure 4b). At the same time, the amount of Hg inside the film, measured by SEM-EDS, dropped from 10% to 1%.

Under visible light in the blue (455 nm), green (530 nm), and red (625 nm), the device obtained with this strategy showed a slow photoresponse (Figures 4c and S1). Blue light illumination yielded the maximum responsivity (0.5 A/W), with a measured detectivity of ca 9 × 10^4^ Jones, whereas red light led to the lowest responsivity (0.1 A/W), in agreement with the absorption spectrum of the material (see Figure S15 for the estimation of the noise spectral power density). The photocurrent had a sublinear dependence on light power (*P*, *I*_pc_ ≈ *P*^0.8^); therefore, the responsivity (*I*_pc_/*P*) was decreasing with *P*, as shown in Figure 4d. This was attributed to the presence of trap states in the material.^54^ Although the performance achieved by our system (*R* = 0.5 A/W and *D* = 9 × 10^4^ Jones) was still far from the best values found in the literature for optimized systems, such as CdTe NCs-P3HT-PCBM (*R* = 50 A/W and *D* = 5 × 10^12^ Jones)^40^ or chlorine-treated CdTe NCs (*R* = 4 × 10^9^ A/W and *D* = 5 × 10^17^ Jones),^42^ it outperformed that of several other systems, such as the thiol-capped CdTe NCs prepared by electrostatic self-assembly (*R* = 0.18 A/W)^41^ or the iodine-passivated CdSe_*x*_Te_1–*x*_ NCs (*R* = 0.053 A/W and *D* = 8 × 10^13^ Jones).^43^ We note that care has to be taken in comparing detectivities because noise evaluation in the literature is not always performed according to the same procedure.^55^

## Conclusions

We synthesized CdTe NCs which were subjected to partial CE with Hg^2+^ cations. This was performed by working at RT and by employing HgCl_2_, a slowly reacting Hg^2+^ precursor, dissolved in methanol. We varied the Hg/Cd feed ratio to prepare three different samples containing 10, 30, or 40% of Hg, respectively. The products of the exchange, CdTe@HgTe core@shell NCs, when annealed at temperatures as low as 200 °C, were observed to evolve in the following way: the superficial HgTe melted or sublimated leading to the sintering of residual CdTe cores and to the concomitant removal of the native surface ligands. The Hg content in the resulting sintered CdTe NC films was found to be much lower than that in the starting CdTe@HgTe NCs, whereas a minor fraction of the extracted Te recrystallized in the form of metallic Te. Given the low sintering temperature, we tested this approach to fabricate proof-of-concept photodetectors based on CdTe thin films which exhibited a photoresponse of up to 0.5 A/W. Considering the high temperatures (above 350 °C) and post-process ligand stripping steps that are currently used in the preparation of CdTe thin films employed in photovoltaic technology, alternative methods aimed at lowering the overall costs of the production of thin films are of great technological importance. In this regard, our novel strategy opens the door to include CE protocols as valid alternatives to just ligand exchange methods for further development of thin-film fabrication at lower temperatures. Clearly, if our procedure were to be employed in the future, it would definitely require an assessment of the correlated environmental risks.

## Supporting Information Available

TEM and HRTEM images, XPS and TEM–EDS analyses, XRD patterns of samples at RT and 200 °C, Raman spectra of TeO_2_ and Te, FTIR spectrum of a KBr pellet, SEM images of a CdTe film obtained by employing CdTe–Hg 10% NCs and the corresponding photocurrent, and plots of the photocurrent as a function of power density at different photoexcitation wavelengths (PDF)

## References

[ref1] Wang H.; Butler D. J.; Straus D. B.; Oh N.; Wu F.; Guo J.; Xue K.; Lee J. D.; Murray C. B.; Kagan C. R. Air-Stable CuInSe_2_ Nanocrystal Transistors and Circuits Via Post-Deposition Cation Exchange. ACS Nano 2019, 13, 2324 10.1021/acsnano.8b09055. 30707549

[ref2] De Trizio L.; Manna L. Forging Colloidal Nanostructures Via Cation Exchange Reactions. Chem. Rev. 2016, 116, 10852–10887. 10.1021/acs.chemrev.5b00739. 26891471 PMC5043423

[ref3] Shamsi J.; Urban A. S.; Imran M.; De Trizio L.; Manna L. Metal Halide Perovskite Nanocrystals: Synthesis, Post-Synthesis Modifications, and Their Optical Properties. Chem. Rev. 2019, 119, 3296 10.1021/acs.chemrev.8b00644. 30758194 PMC6418875

[ref4] Rivest J. B.; Jain P. K. Cation Exchange on the Nanoscale: An Emerging Technique for New Material Synthesis, Device Fabrication, and Chemical Sensing. Chem. Soc. Rev. 2013, 42, 89–96. 10.1039/C2CS35241A. 22968228

[ref5] Son D. H.; Hughes S. M.; Yin Y.; Alivisatos A. P. Cation Exchange Reactions in Ionic Nanocrystals. Science 2004, 306, 1009–1012. 10.1126/science.1103755. 15528440

[ref6] Moon G. D.; Ko S.; Min Y.; Zeng J.; Xia Y.; Jeong U. Chemical Transformations of Nanostructured Materials. Nano Today 2011, 6, 186–203. 10.1016/j.nantod.2011.02.006.

[ref7] Fayette M.; Robinson R. D. Chemical Transformations of Nanomaterials for Energy Applications. J. Mater. Chem. A 2014, 2, 5965 10.1039/c3ta13982d.

[ref8] Gupta S.; Kershaw S. V.; Rogach A. L. 25th Anniversary Article: Ion Exchange in Colloidal Nanocrystals. Adv. Mater. 2013, 25, 6923–6944. 10.1002/adma.201302400. 24108549

[ref9] van der Stam W.; Geuchies J. J.; Altantzis T.; van den Bos K. H. W.; Meeldijk J. D.; Van Aert S.; Bals S.; Vanmaekelbergh D.; de Mello Donega C. Highly Emissive Divalent-Ion-Doped Colloidal CsPb_1–x_M_x_Br_3_ Perovskite Nanocrystals through Cation Exchange. J. Am. Chem. Soc. 2017, 139, 4087–4097. 10.1021/jacs.6b13079. 28260380 PMC5364419

[ref10] Tu R.; Xie Y.; Bertoni G.; Lak A.; Gaspari R.; Rapallo A.; Cavalli A.; Trizio L. D.; Manna L. Influence of the Ion Coordination Number on Cation Exchange Reactions with Copper Telluride Nanocrystals. J. Am. Chem. Soc. 2016, 138, 7082–7090. 10.1021/jacs.6b02830. 27177274 PMC5736242

[ref11] Li H.; Zanella M.; Genovese A.; Povia M.; Falqui A.; Giannini C.; Manna L. Sequential Cation Exchange in Nanocrystals: Preservation of Crystal Phase and Formation of Metastable Phases. Nano Lett. 2011, 11, 4964–4970. 10.1021/nl202927a. 21961554

[ref12] De Trizio L.; Gaspari R.; Bertoni G.; Kriegel I.; Moretti L.; Scotognella F.; Maserati L.; Zhang Y.; Messina G. C.; Prato M.; Marras S.; Cavalli A.; Manna L. Cu_3-x_P Nanocrystals as a Material Platform for near-Infrared Plasmonics and Cation Exchange Reactions. Chem. Mater. 2015, 27, 1120–1128. 10.1021/cm5044792. 25960605 PMC4419285

[ref13] Powell A. E.; Hodges J. M.; Schaak R. E. Preserving Both Anion and Cation Sublattice Features During a Nanocrystal Cation-Exchange Reaction: Synthesis of Metastable Wurtzite-Type Cos and Mns. J. Am. Chem. Soc. 2016, 138, 471–474. 10.1021/jacs.5b10624. 26689081

[ref14] Grodzińska D.; Pietra F.; van Huis M. A.; Vanmaekelbergh D.; de Mello Donegá C. Thermally Induced Atomic Reconstruction of PbSe/CdSe Core/Shell Quantum Dots into PbSe/CdSe Bi-Hemisphere Hetero-Nanocrystals. J. Mater. Chem. 2011, 21, 11556–11565. 10.1039/C0JM04458J.

[ref15] Wang X.; Liu X.; Zhu D.; Swihart M. T. Controllable Conversion of Plasmonic Cu_2–x_S Nanoparticles to Au_2_S by Cation Exchange and Electron Beam Induced Transformation of Cu_2–x_S–Au_2_S Core/Shell Nanostructures. Nanoscale 2014, 6, 8852–8857. 10.1039/C4NR02114B. 24957012

[ref16] Gariano G.; Lesnyak V.; Brescia R.; Bertoni G.; Dang Z.; Gaspari R.; De Trizio L.; Manna L. Role of the Crystal Structure in Cation Exchange Reactions Involving Colloidal Cu_2_Se Nanocrystals. J. Am. Chem. Soc. 2017, 139, 9583–9590. 10.1021/jacs.7b03706. 28644018 PMC6105078

[ref17] Balcerak R.; Gibson J. F.; Gutierrez W. A.; Pollard J. H. Evolution of a New Semiconductor Product: Mercury Cadmium Telluride Focal Plane Arrays. Opt. Eng. 1987, 26, 263191 10.1117/12.7974050.

[ref18] Yang J.; Zhou Y.; Zheng S.; Liu X.; Qiu X.; Tang Z.; Song R.; He Y.; Ahn C. W.; Kim J. W. Self-Reorganization of CdTe Nanoparticles into Near-Infrared Hg_1–x_Cd_x_Te Nanowire Networks. Chem. Mater. 2009, 21, 3177–3182. 10.1021/cm900625w.

[ref19] Smith A. M.; Nie S. Bright and Compact Alloyed Quantum Dots with Broadly Tunable Near-Infrared Absorption and Fluorescence Spectra through Mercury Cation Exchange. J. Am. Chem. Soc. 2011, 133, 24–26. 10.1021/ja108482a. 21142154 PMC3102112

[ref20] Rogach A. L.; Harrison M. T.; Kershaw S. V.; Kornowski A.; Burt M. G.; Eychmüller A.; Weller H. Colloidally Prepared CdHgTe and HgTe Quantum Dots with Strong near-Infrared Luminescence. Phys. Status Solidi B 2001, 224, 153–158. 10.1002/1521-3951(200103)224:1<153::aid-pssb153>3.0.co;2-3.

[ref21] Shen G.; Guyot-Sionnest P. HgTe/CdTe and HgSe/CdX (X = S, Se, and Te) Core/Shell Mid-Infrared Quantum Dots. Chem. Mater. 2019, 31, 286–293. 10.1021/acs.chemmater.8b04727.

[ref22] Wang H.; Lou S.; Tang Z.; Xu W.; Shang H.; Shen H.; Li L. S. Thiolate-Assisted Cation Exchange Reaction for the Synthesis of near-Infrared Photoluminescent Hg_x_Cd_1–x_Te Nanocrystals. Dalton Trans. 2012, 41, 12726–12732. 10.1039/C2DT31602A. 22968476

[ref23] Smith A. M.; Lane L. A.; Nie S. Mapping the Spatial Distribution of Charge Carriers in Quantum-Confined Heterostructures. Nat. Commun. 2014, 5, 4506 10.1038/ncomms5506. 25080298 PMC4122291

[ref24] Gupta S.; Zhovtiuk O.; Vaneski A.; Lin Y.-C.; Chou W.-C.; Kershaw S. V.; Rogach A. L. Cd_x_Hg_(1–x)_Te Alloy Colloidal Quantum Dots: Tuning Optical Properties from the Visible to near-Infrared by Ion Exchange. Part. Part. Syst. Char. 2013, 30, 346–354. 10.1002/ppsc.201200139.

[ref25] Szofran F. R.; Lehoczky S. L. The Pseudobinary HgTe-CdTe Phase Diagram. J. Electron. Mater. 1981, 10, 1131–1150. 10.1007/bf02661194.

[ref26] Kershaw S. V.; Burt M.; Harrison M.; Rogach A.; Weller H.; Eychmüller A. Colloidal CdTe/HgTe Quantum Dots with High Photoluminescence Quantum Efficiency at Room Temperature. Appl. Phys. Lett. 1999, 75, 1694–1696. 10.1063/1.124792.

[ref27] Choi D.; Yoon B.; Kim D.-K.; Baik H.; Choi J.-H.; Jeong K. S. Major Electronic Transition Shift from Bandgap to Localized Surface Plasmon Resonance in Cd_x_Hg_1–x_Se Alloy Nanocrystals. Chem. Mater. 2017, 29, 8548–8554. 10.1021/acs.chemmater.7b03813.

[ref28] Harrison M. T.; Kershaw S. V.; Burt M. G.; Eychmüller A.; Weller H.; Rogach A. L. Wet Chemical Synthesis and Spectroscopic Study of CdHgTe Nanocrystals with Strong Near-Infrared Luminescence. J. Mater. Sci. Eng. B 2000, 69–70, 355–360. 10.1016/s0921-5107(99)00254-8.

[ref29] Izquierdo E.; Robin A.; Keuleyan S.; Lequeux N.; Lhuillier E.; Ithurria S. Strongly Confined HgTe 2d Nanoplatelets as Narrow near-Infrared Emitters. J. Am. Chem. Soc. 2016, 138, 10496–10501. 10.1021/jacs.6b04429. 27487074

[ref30] Gréboval C.; Izquierdo E.; Livache C.; Martinez B.; Dufour M.; Goubet N.; Moghaddam N.; Qu J.; Chu A.; Ramade J.; Aubin H.; Cruguel H.; Silly M.; Lhuillier E.; Ithurria S. Impact of Dimensionality and Confinement on the Electronic Properties of Mercury Chalcogenide Nanocrystals. Nanoscale 2019, 11, 3905 10.1039/C8NR09644A. 30758021

[ref31] Izquierdo E.; Dufour M.; Chu A.; Livache C.; Martinez B.; Amelot D.; Patriarche G.; Lequeux N.; Lhuillier E.; Ithurria S. Coupled HgSe Colloidal Quantum Wells through a Tunable Barrier: A Strategy to Uncouple Optical and Transport Band Gap. Chem. Mater. 2018, 30, 4065–4072. 10.1021/acs.chemmater.8b01028.

[ref32] Clayton A. J.; Barrioz V. Chapter 5 Thin Film Cadmium Telluride Solar Cells. Materials Challenges: Inorganic Photovoltaic Solar Energy; The Royal Society of Chemistry, 2015; pp 135–159.

[ref33] Polman A.; Knight M.; Garnett E. C.; Ehrler B.; Sinke W. C. Photovoltaic Materials: Present Efficiencies and Future Challenges. Science 2016, 352, aad4424 10.1126/science.aad4424. 27081076

[ref34] Zhang H.; Kurley J. M.; Russell J. C.; Jang J.; Talapin D. V. Solution-Processed, Ultrathin Solar Cells from CdCl_3_^-^-Capped CdTe Nanocrystals: The Multiple Roles of CdCl_3_^-^ Ligands. J. Am. Chem. Soc. 2016, 138, 7464–7467. 10.1021/jacs.6b03240. 27269672

[ref35] Crisp R. W.; Panthani M. G.; Rance W. L.; Duenow J. N.; Parilla P. A.; Callahan R.; Dabney M. S.; Berry J. J.; Talapin D. V.; Luther J. M. Nanocrystal Grain Growth and Device Architectures for High-Efficiency CdTe Ink-Based Photovoltaics. ACS Nano 2014, 8, 9063–9072. 10.1021/nn502442g. 25133302

[ref36] Kagan C. R.; Lifshitz E.; Sargent E. H.; Talapin D. V. Building Devices from Colloidal Quantum Dots. Science 2016, 353, aac5523 10.1126/science.aac5523. 27563099

[ref37] MacDonald B. I.; Martucci A.; Rubanov S.; Watkins S. E.; Mulvaney P.; Jasieniak J. J. Layer-by-Layer Assembly of Sintered CdSe_x_Te_1–x_ Nanocrystal Solar Cells. ACS Nano 2012, 6, 5995–6004. 10.1021/nn3009189. 22690798

[ref38] Townsend T. K.; Heuer W. B.; Foos E. E.; Kowalski E.; Yoon W.; Tischler J. G. Safer Salts for Cdte Nanocrystal Solution Processed Solar Cells: The Dual Roles of Ligand Exchange and Grain Growth. J. Mater. Chem. A 2015, 3, 13057–13065. 10.1039/C5TA02488A.

[ref39] Panthani M. G.; Kurley J. M.; Crisp R. W.; Dietz T. C.; Ezzyat T.; Luther J. M.; Talapin D. V. High Efficiency Solution Processed Sintered CdTe Nanocrystal Solar Cells: The Role of Interfaces. Nano Lett. 2014, 14, 670–675. 10.1021/nl403912w. 24364381

[ref40] Chen H.-Y.; Lo M. K. F.; Yang G.; Monbouquette H. G.; Yang Y. Nanoparticle-Assisted High Photoconductive Gain in Composites of Polymer and Fullerene. Nat. Nanotechnol. 2008, 3, 543–547. 10.1038/nnano.2008.206. 18772915 PMC3966304

[ref41] Tu C.-C.; Lin L. Y. High Efficiency Photodetectors Fabricated by Electrostatic Layer-by-Layer Self-Assembly of CdTe Quantum Dots. Appl. Phys. Lett. 2008, 93, 163107 10.1063/1.3003883.

[ref42] Zhang Y.; Hellebusch D. J.; Bronstein N. D.; Ko C.; Ogletree D. F.; Salmeron M.; Alivisatos A. P. Ultrasensitive Photodetectors Exploiting Electrostatic Trapping and Percolation Transport. Nat. Commun. 2016, 7, 11924 10.1038/ncomms11924. 27323904 PMC4919514

[ref43] Shen T.; Li B.; Zheng K.; Pullerits T.; Cao G.; Tian J. Surface Engineering of Quantum Dots for Remarkably High Detectivity Photodetectors. J. Phys. Chem. Lett. 2018, 9, 3285–3294. 10.1021/acs.jpclett.8b01255. 29862824

[ref44] Miszta K.; Gariano G.; Brescia R.; Marras S.; De Donato F.; Ghosh S.; De Trizio L.; Manna L. Selective Cation Exchange in the Core Region of Cu_2–x_Se/Cu_2–x_S Core/Shell Nanocrystals. J. Am. Chem. Soc. 2015, 137, 12195–12198. 10.1021/jacs.5b06379. 26360611 PMC4591528

[ref45] Taniguchi S.; Green M.; Lim T. The Room-Temperature Synthesis of Anisotropic Cdhgte Quantum Dot Alloys: A “Molecular Welding” Effect. J. Am. Chem. Soc. 2011, 133, 3328–3331. 10.1021/ja200132d. 21332163

[ref46] Ringel S. A.; Smith A. W.; MacDougal M. H.; Rohatgi A. The Effects of CdCl_2_ on the Electronic Properties of Molecular-Beam Epitaxially Grown CdTe/CdS Heterojunction Solar Cells. J. Appl. Phys. 1991, 70, 881–889. 10.1063/1.349652.

[ref47] Major J. D.; Treharne R. E.; Phillips L. J.; Durose K. A Low-Cost Non-Toxic Post-Growth Activation Step for CdTe Solar Cells. Nature 2014, 511, 334 10.1038/nature13435. 25030171

[ref48] Maniscalco B.; Abbas A.; Bowers J. W.; Kaminski P. M.; Bass K.; West G.; Walls J. M. The Activation of Thin Film CdTe Solar Cells Using Alternative Chlorine Containing Compounds. Thin Solid Films 2015, 582, 115–119. 10.1016/j.tsf.2014.10.059.

[ref49] Dharmadasa I. M.; Echendu O. K.; Fauzi F.; Abdul-Manaf N. A.; Olusola O. I.; Salim H. I.; Madugu M. L.; Ojo A. A. Improvement of Composition of CdTe Thin Films During Heat Treatment in the Presence of CdCl_2_. J. Mater. Sci.: Mater. Electron. 2017, 28, 2343–2352. 10.1007/s10854-016-5802-9.

[ref50] Atzmüller R.; Rösch M.; Schaack G.; Becker C. R. Quantum Confinement Effects above the Fundamental Band Gap in HgTe/Hg_0.3_Cd_0.7_Te Heterostructures Studied by Resonant Raman Scattering near the E_1_ Edge. Phys. Rev. B: Condens. Matter Mater. Phys. 1996, 54, 16907–16918. 10.1103/PhysRevB.54.16907. 9985819

[ref51] Ren A.; Liu C.; Gao W.; Wang F.; Liu Y.; Wu L.; Wang W.; Li W.; Zhang J.; Feng L. Characterization and Annealing of CdTe Thin Film Prepared by Vapor Transport Deposition. Chalcogenide Lett. 2015, 12, 555–567.

[ref52] Dharmadasa I. Review of the CdCl_2_ Treatment Used in CdS/CdTe Thin Film Solar Cell Development and New Evidence Towards Improved Understanding. Coatings 2014, 4, 282 10.3390/coatings4020282.

[ref53] Qi W. H.; Wang M. P. Size Effect on the Cohesive Energy of Nanoparticle. J. Mater. Sci. Lett. 2002, 21, 1743–1745. 10.1023/A:1020904317133.

[ref54] Taylor G. W.; Simmons J. G. Photoconductivity Characteristics of Defect Insulators. J. Phys. C: Solid State Phys. 1975, 8, 3360–3370. 10.1088/0022-3719/8/20/014.

[ref55] Fang Y.; Armin A.; Meredith P.; Huang J. Accurate Characterization of Next-Generation Thin-Film Photodetectors. Nat. Photonics 2019, 13, 1–4. 10.1038/s41566-018-0288-z.

