# Supplementary material for: Metastable CdTe@HgTe Core@Shell Nanostructures Obtained by Partial Cation Exchange Evolve into Sintered CdTe Films Upon Annealing
Source: Chem Mater. 2020 Mar 12;32(7):2978–85. doi: 10.1021/acs.chemmater.9b05281 (PMC8016170; doi:10.1021/acs.chemmater.9b05281)
Supplement: Supplementary file 1 — cm9b05281_si_001.pdf [file cm9b05281_si_001.pdf]

SUPPORTING INFORMATION FOR

Metastable CdTe@HgTe core@shell Nanostructures Obtained by Partial Cation Exchange Evolve into Sintered CdTe films Upon Annealing

Irene Rosina[a,b], Beatriz Martin-Garcia[c], Davide Spirito[d], Zhiya Dang[a], Graziella Gariano[a], Sergio Marras[e], Mirko Prato[e], Roman Krahne[d], Luca De Trizio[a] and Liberato Manna[a]

[a]Nanochemistry Department, [c]Graphene Labs, [d]Optoelectronics, [e]Materials Characterization Facility, Istituto Italiano di Tecnologia (IIT), via Morego 30, Genova, Italy

[b]Dipartimento di Chimica e Chimica Industriale, Università degli Studi di Genova, Via Dodecaneso 31, 16146 Genova, Italy

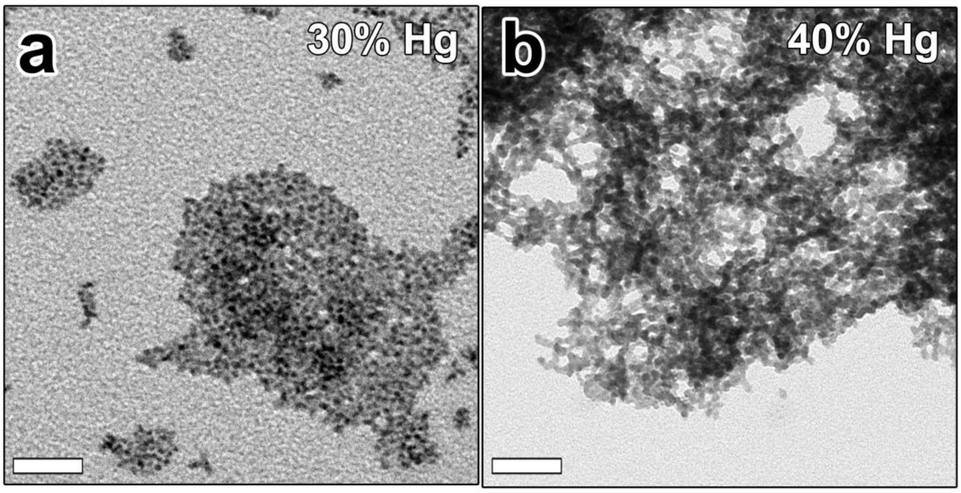

Figure S1. TEM images of CdTe-Hg NCs obtained by partial CE with Hg^{2+} cations employing Hg/Cd feed ratios of (a) 30% and (b) 40%. The scalebars are 50nm.

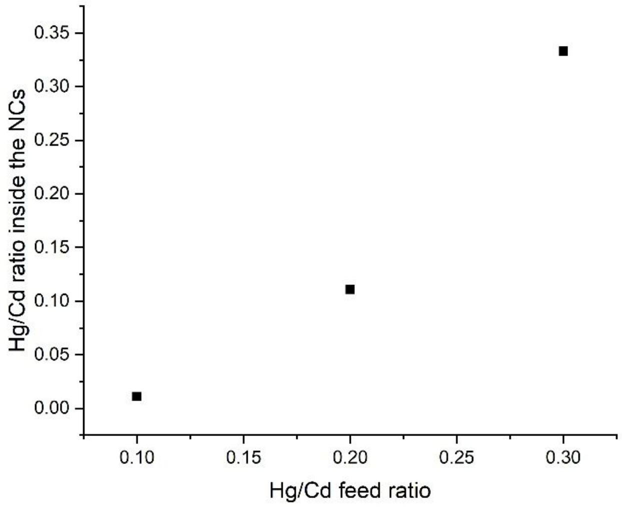

Figure S2. Hg/Cd ratio inside the NCs (measured via ICP analysis) as a function of the Hg/Cd feed ratio employed in the CE experiments.

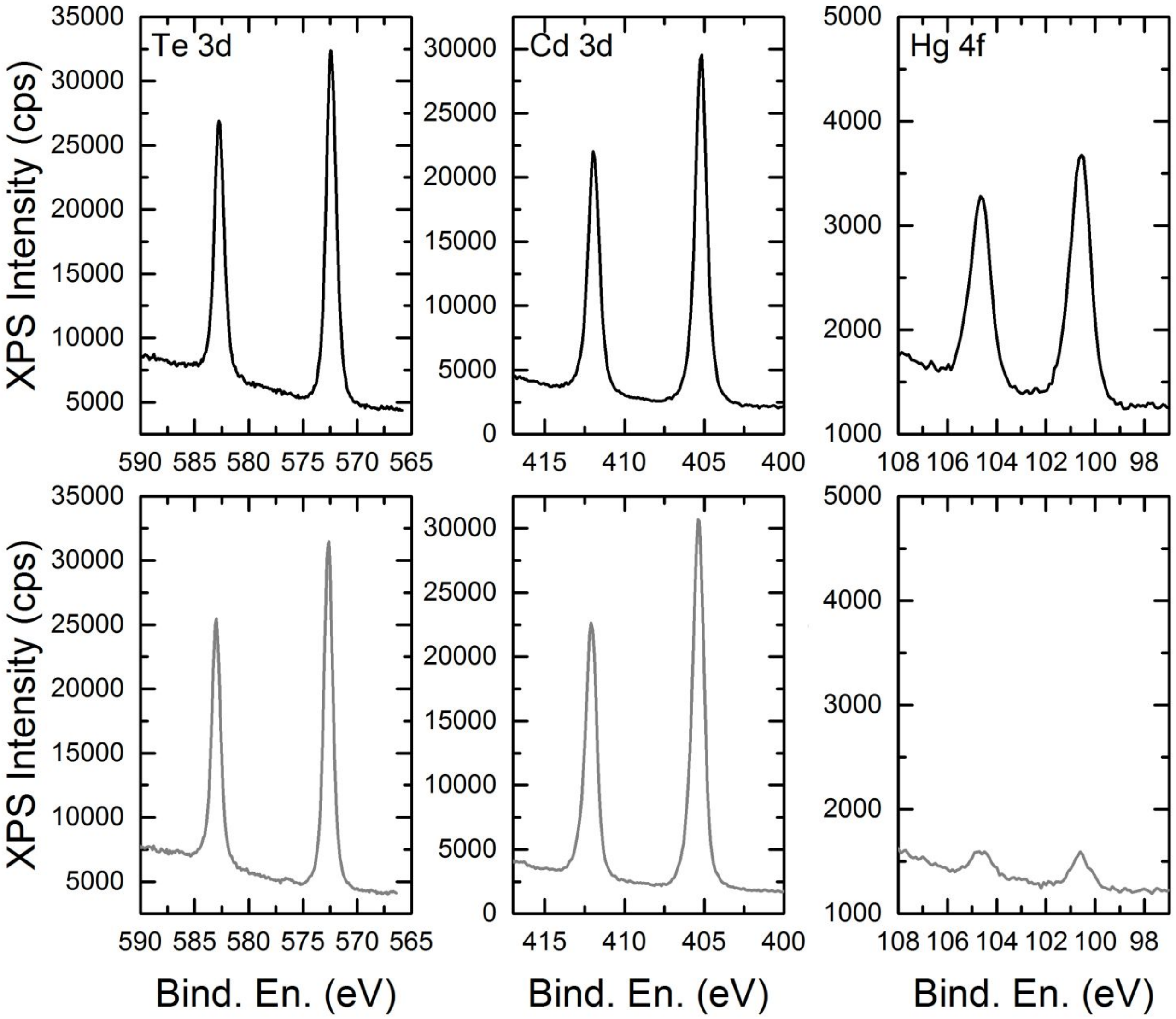

Figure S3. XPS spectra of CdTe-Hg 10% NCs before (upper panels) and after annealing (lower panels). Peak positions: Te $3d_{5/2}$ at (572.5 ± 0.2) eV, Cd $3d_{5/2}$ at (405.3 ± 0.2) eV and Hg $4f_{7/2}$ at (100.6 ± 0.2) eV, indicating the presence of Te^{2-}, Cd^{2+} and Hg^{2+} in the sample [NIST X-ray Photoelectron Spectroscopy Database, Version 4.1 (National Institute of Standards and Technology, Gaithersburg, 2012); http://srdata.nist.gov/xps/]. Relative atomic ratios were determined using the integrated area under Cd 3d, Te 3d and Hg 4f principal peaks.

Computational model:

A zincblende $Cd_{1028}Te_{919}$ nanocrystal of ~4.2nm in diameter has been constructed. This model features a Cd/Se ratio of 1.12 and exposes four Te rich ($\overline{1}\overline{1}\overline{1}$), four Cd rich (111), and six Cd rich (100) facets (see Figure SXX). By replacing all the superficial Cd atoms by Hg ones, the stoichiometry becomes $Cd_{724}Hg_{304}Te_{919}$,. Our model indicates that a HgTe monolayer can be achieved by CE only when substituting almost 40% of Cd^{2+} cations that is, when achieving Hg/Cd ratio of 0.42.

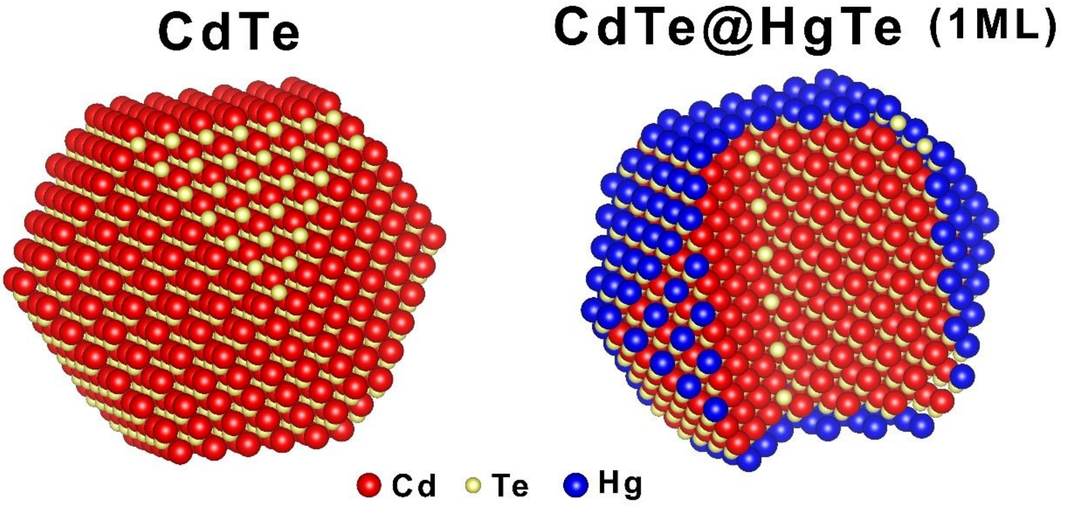

Figure S4. Models of zincblende 4.2 nm CdTe and CdTe@HgTe nanocrystals, the latter being characterized by one HgTe monolayer thick shell.

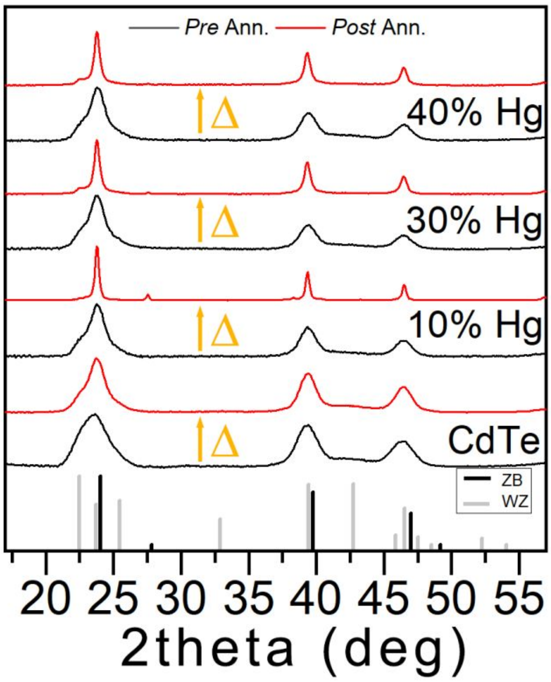

Figure S5. XRD patterns of CdTe and CdTe-Hg NC samples subjected to a thermal treatment under N_2 from RT (black curves) to 200°C (red curves). The bulk reflections of WZ and ZB CdTe structures are reported by means of gray and black bars, respectively.

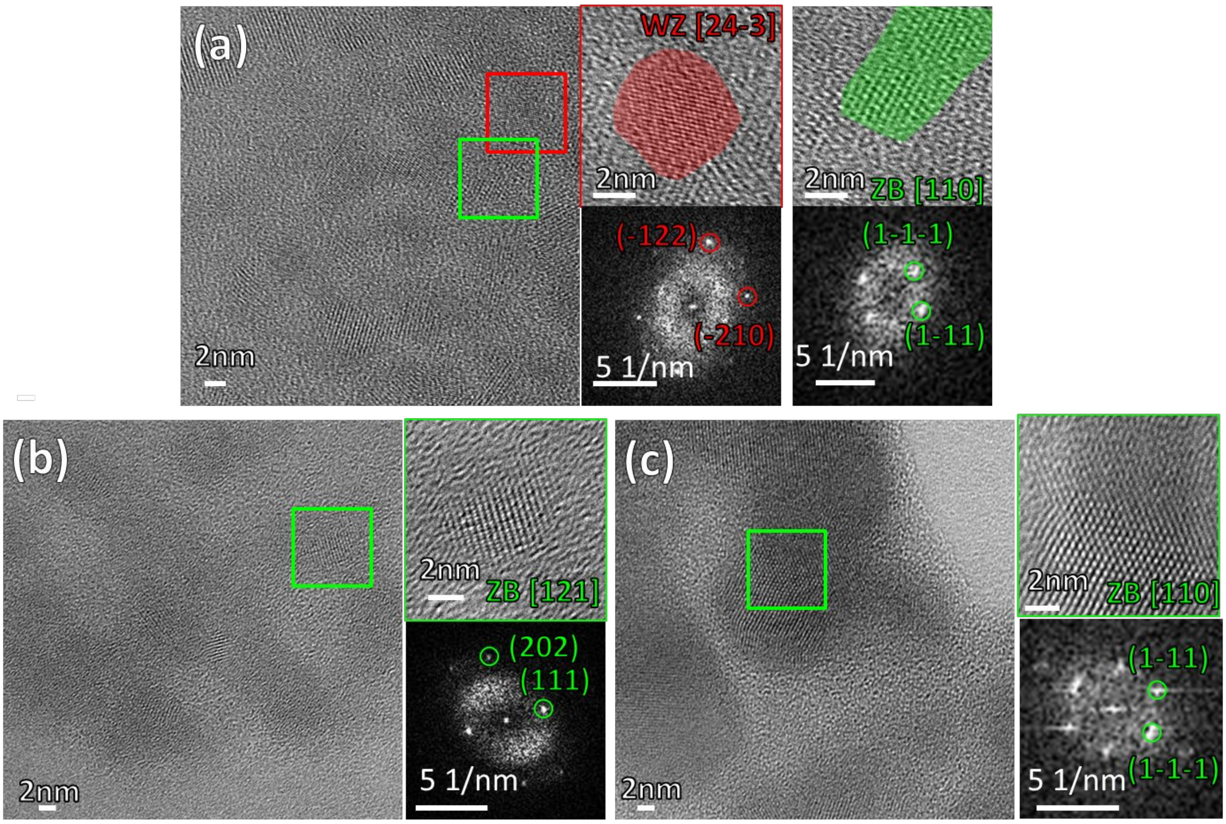

Figure S6. HRTEM images of CdTe-Hg 10% NCs before (a) and after annealing at 200°C (b,c) with the corresponding FFT.

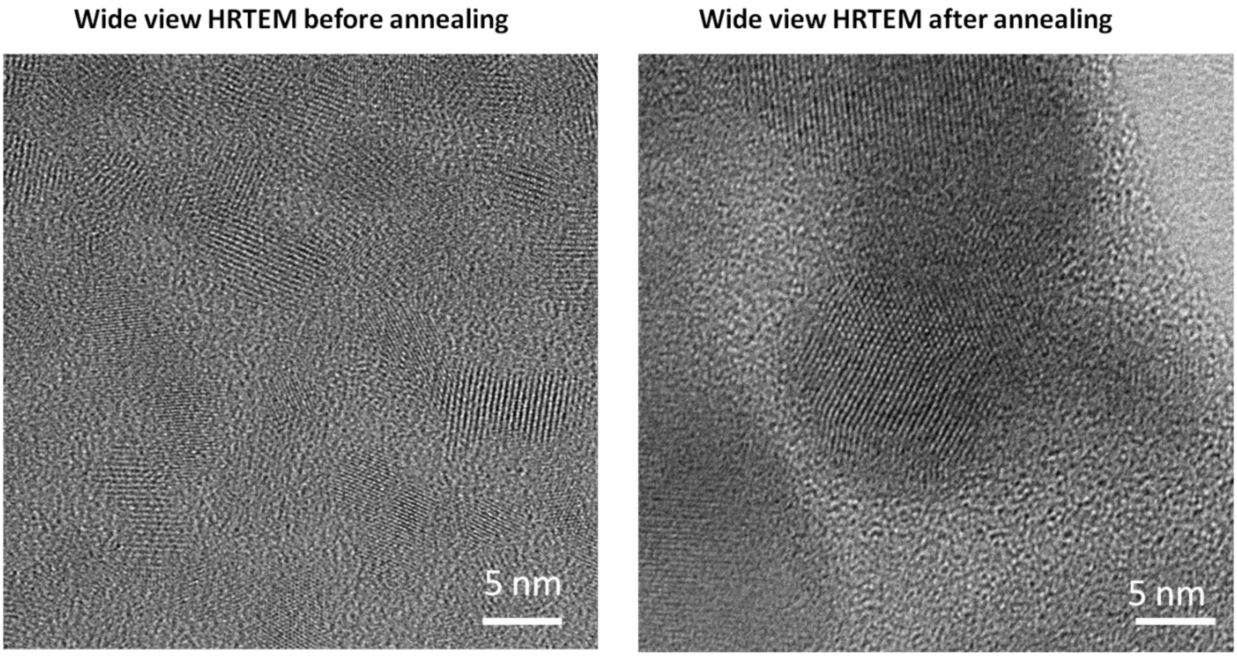

Figure S7. Wide view HRTEM images of CdTe-Hg 10% NCs before (left) and after (right) annealing, showing that NCs have merged into larger crystallites.

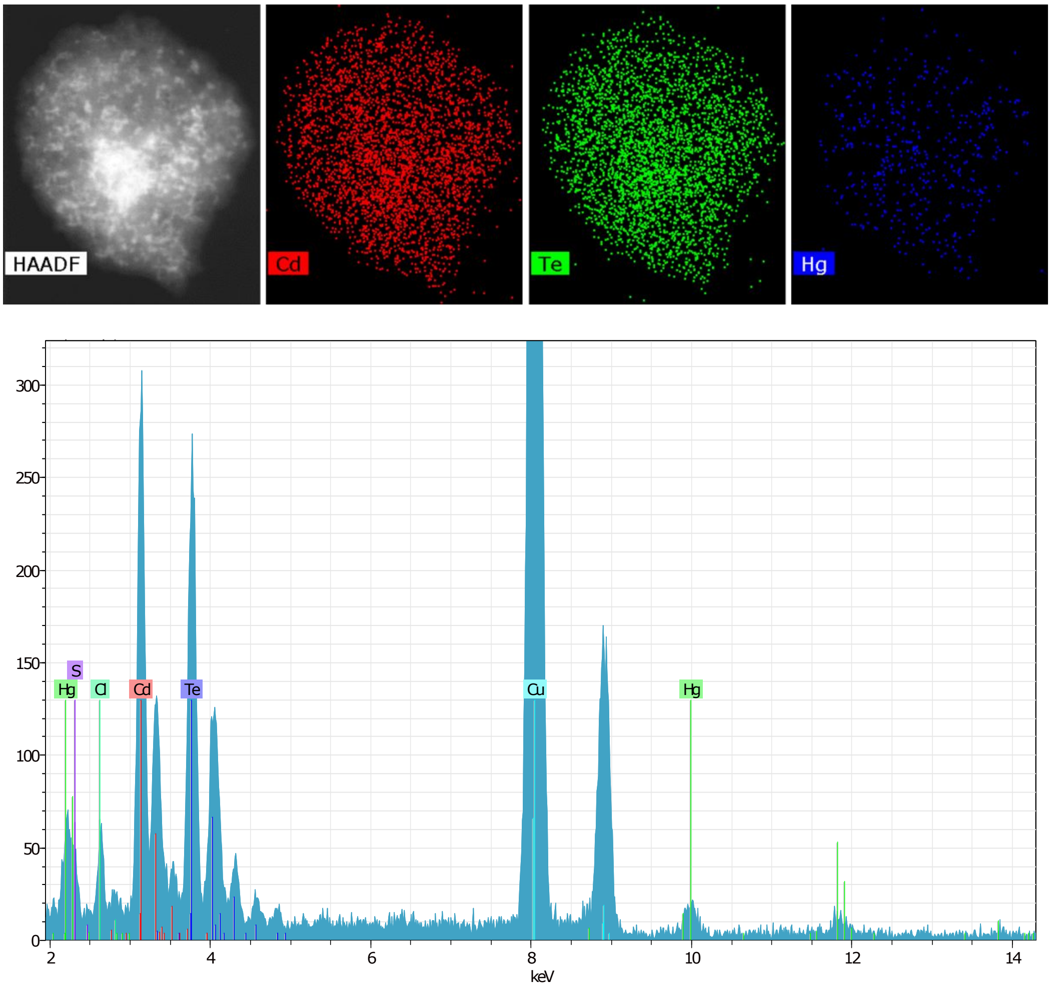

Figure S8. STEM-EDX mapping of CdTe@HgTe 10% NCs (top panels) with the corresponding measured spectrum (bottom panel). The composition of the NC ensemble was measured to be $CdHg_{0.1}Te$.

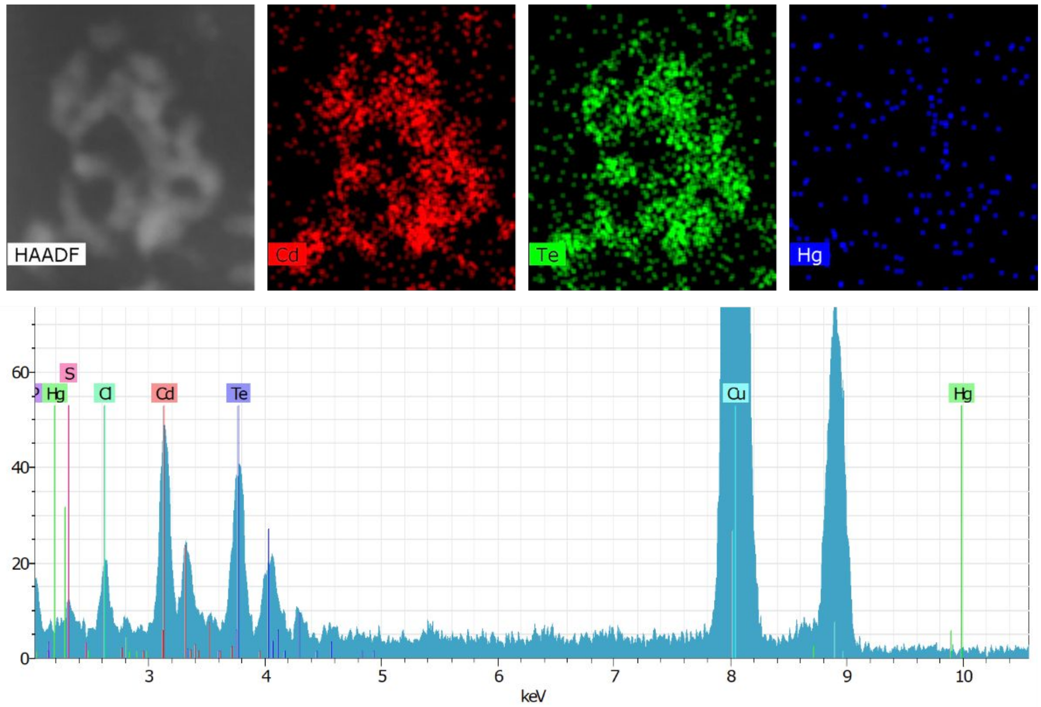

Figure S9. STEM-EDX mapping of CdTe-Hg 10% NCs after annealing at 200°C (top panels) with the corresponding spectrum (bottom panel). The composition of the NC ensemble was measured to be $Cd_{1.03}Te$.

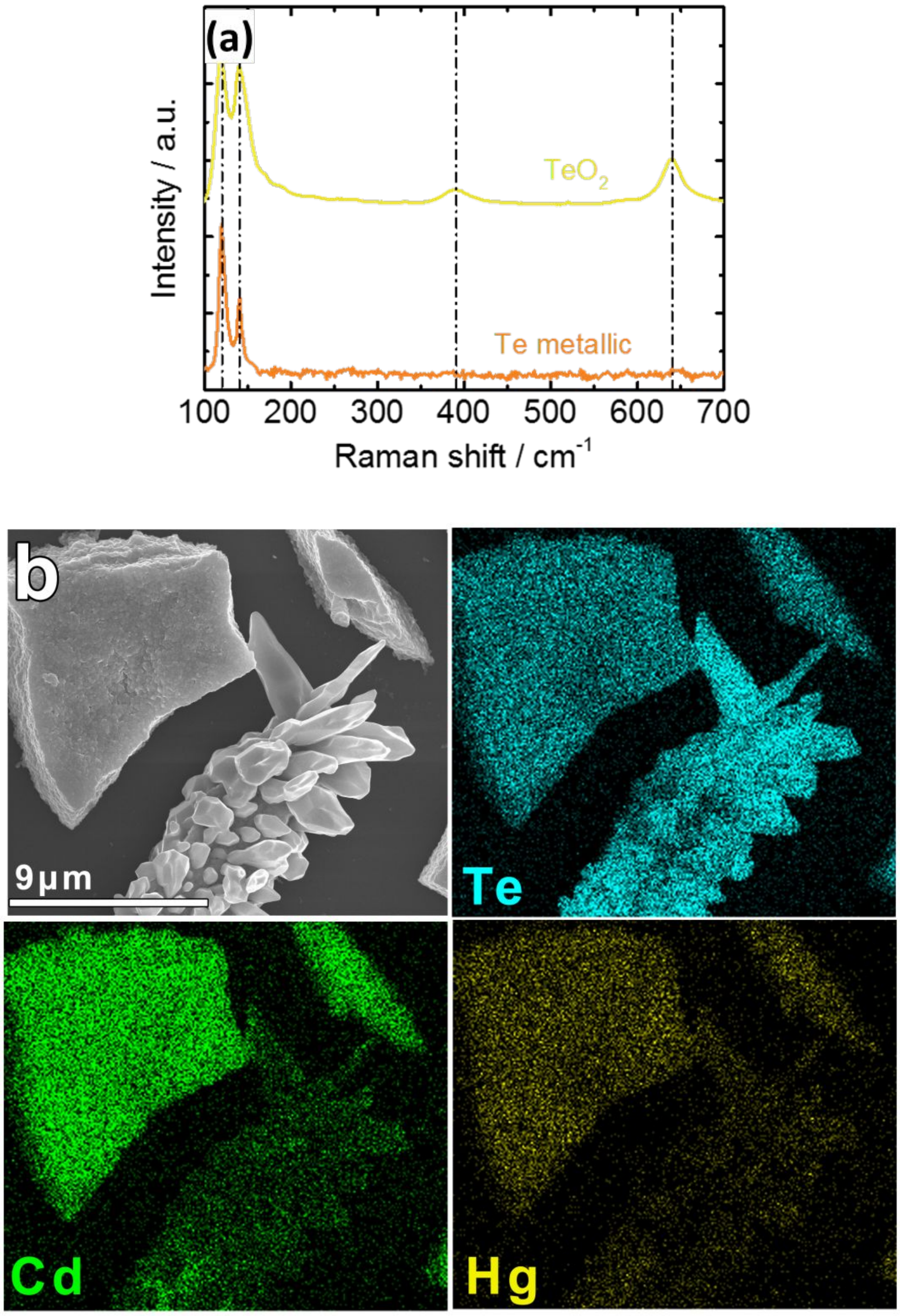

Figure S10. (a) Raman spectra of metallic Te characterized by two peaks at 120 and 140 cm^{-1} and TeO_2 with additional peaks at 390 and 640 cm^{-1}. (b) SEM image of a section of a film of CdTe-Hg 10% NCs after annealing at 200°C. The rod like structure present in the SEM image is composed of Te only, as emerged from the corresponding SEM-EDX measurements.

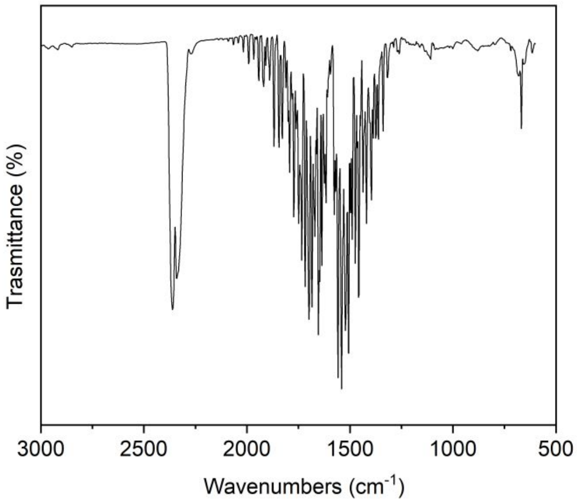

Figure S11. FTIR spectrum of a “blank” KBr pellet employed in the FTIR analyses of CdTe and CdTe-Hg NC samples.

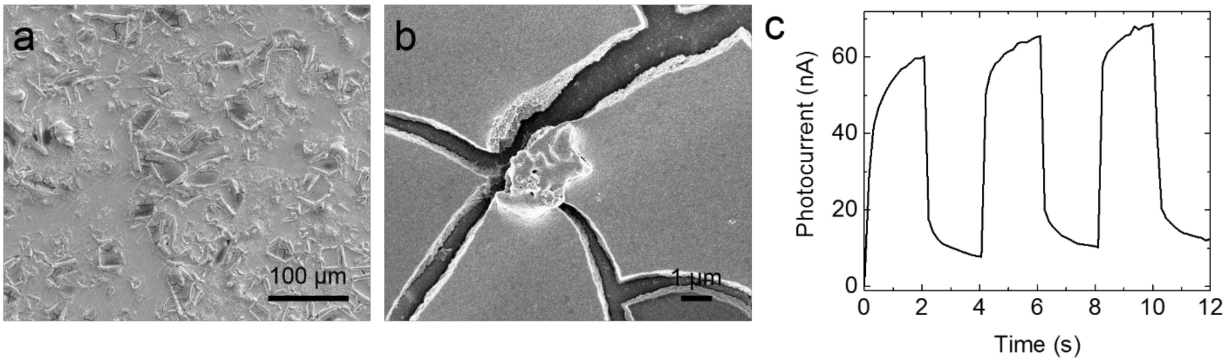

Figure S12. (a, b) Representative SEM images of a CdTe film obtained by spin coating CdTe-Hg 10% NCs and by annealing at 200°C. (c) Time trace of the photocurrent while the light was switched on and off, at room temperature in air under white light (LED ILH-ON09-VA, 90 mW/cm^2).

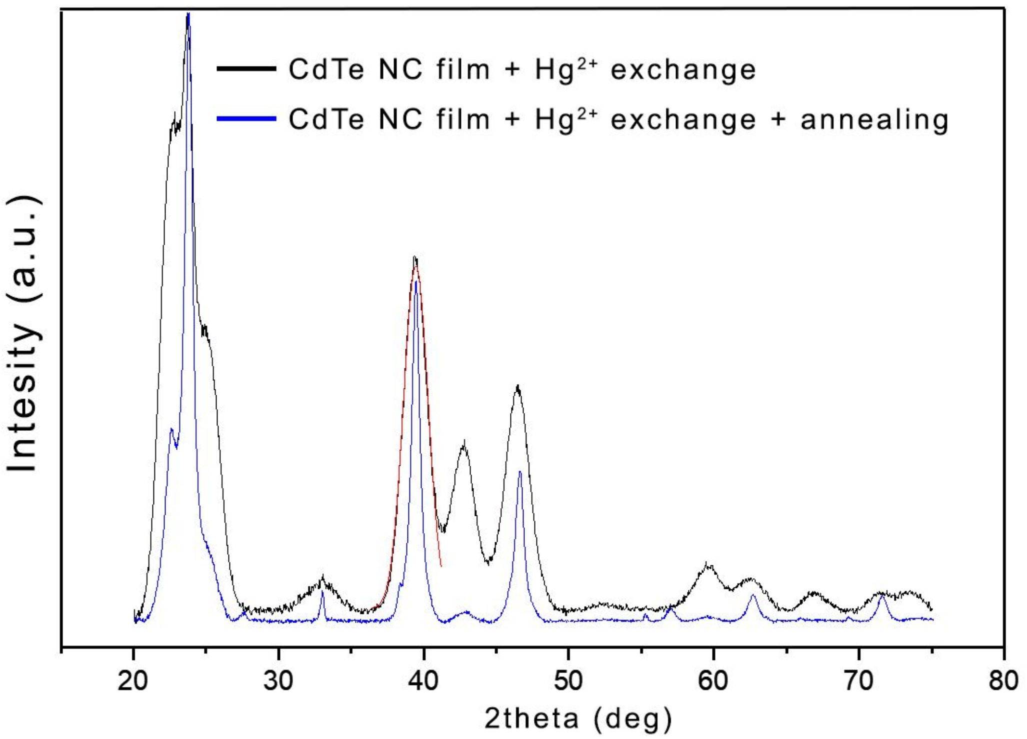

Figure S13. (a) XRD pattern of a film obtained by first depositing CdTe NCs and then by CE directly on the film before (black line) and after (blue line) the annealing step at 200°C. The amount of Hg after the exchange directly on the film, as measured by both ICP-OES and SEM-EDS was 10%, while after the annealing it was ~1%.

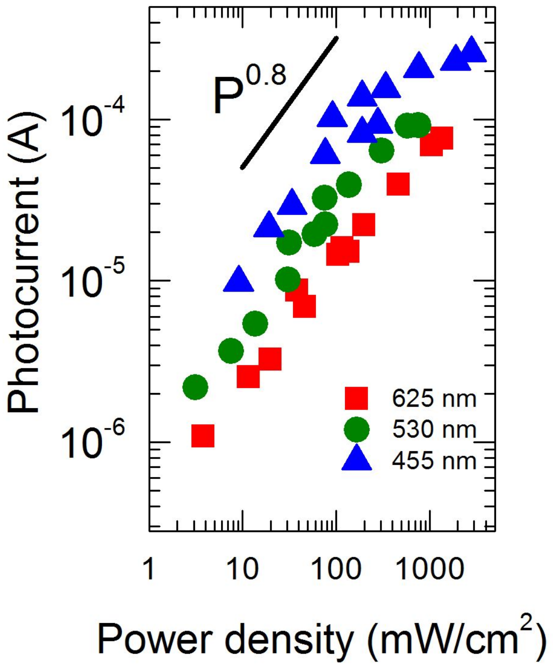

Figure S14. Photocurrent as a function of power density at different photoexcitation wavelengths: 455 nm (blue), 530 nm (green) and 625 nm (red). The continuous, black line marks power law dependence $P^{0.8}$.

Detectivity estimation

The best theoretical value for the detectivity can be estimated from the lower limit to the noise, calculated including thermal and shot noise. The noise spectral density in these cases are:

$$S_{th} = \sqrt{\frac{k_B T}{R_{dark}}}$$

$$S_{shot} = \sqrt{2eI_{dark}}$$

Where R_{dark} and I_{dark} are dark resistance and current respectively, T is the temperature, k_B and e the Boltzmann constant and elementary charge, respectively.

Noise equivalent power, for the different contributions, can be calculated as the ratio of the spectral density to the responsivity:

$$NEP_i = \frac{S_i}{\mathcal{R}}$$

Finally, the detectivity is given by

$$D^* = \frac{\sqrt{A}}{\sum_i NEP_i}$$

Where A is the device area.

In our device, we have dark current of 1.1 mA at 10 V, resulting in $NEP_{tot} = 3.7\times10^{11}$ W $Hz^{-1/2}$ at the maximum responsivity with blue light.

However, an experimental estimation would be more reliable. In this work, we used a numerical estimation of the noise from a current-time signal acquired in the dark, using the same acquisition setup and during the same measurement run of the photocurrent measurements. This procedure avoids the use of additional instruments that may change the noise performance of the whole system. Furthermore, this procedure is also adequate to estimate the noise at the low frequency, of operation of our device. The noise power spectral density is calculated via Fast Fourier Transform (FFT, see e.g., Allen, Mills, "Signal Analysis: Time, Frequency, Scale, and Structure", 2004, chap. 9). The result is show in the Figure S15, together with the result of the Welch's average periodogram method for comparison (implemented in the psd package of the Python package matplotlib v. 3.1.2, doi: 10.5281/zenodo.3563226).

It can be observed that the noise reaches the flat (white) noise floor at very low frequencies, suggesting that, besides the 1/f noise in the device, also the setup is contributing to the noise. With the resulting spectral noise density, estimating a value of $\sim2\times10^{-7}$ A $Hz^{-1/2}$ at low frequency in our device we have a NEP of 4×10^{-7} W $Hz^{-1/2}$ (blue light) and 10×10^{-7} W $Hz^{-1/2}$, corresponding to a detectivity of $\sim9\times10^{4}$ Jones and 2×10^{4} Jones for blue and red light, respectively.

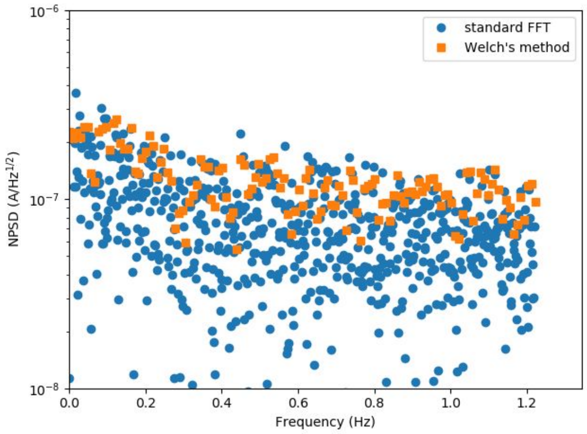

Figure S15. Noise power spectral density (NSPD) for a current-time acquisition, calculated from the FFT or with the Welch's periodogram method.
